# In Memoriam—P. P. Wells, M. D.

**Published:** 1892-01

**Authors:** B. Fincke

**Affiliations:** President


### IN MEMORIAM—P. P. WELLS, M. D.

At a meeting of the New York Homœopathic Union, held December 17th, 1891, the following resolutions were adopted :

WHEREAS, Dr. P. P. Wells, of Brooklyn, N. Y., departed this life November 23d, in his eighty-fourth year, and with him one of our best members and originators of the New York Homœopathic Union has been removed, therefore :

*Resolved*, That we acknowledge with pride and gratitude the great work of his scientific and practical labors in the homœopathic cause.

*Resolved*, That we lose in him a beloved and revered teacher and leader whose name will go down to the latest generations as one of the pioneers of Homœopathy in America.

*Resolved*, That in honor of his memory these resolutions be placed on the records of the Homœopathic Union and published in the homœopathic journals.

*Resolved*, That a copy of these resolutions be presented to the family of our deceased member as a token of our sympathy and respect.

B. FINCKE, M. D., *President*.

